# Evaluation du traitement antirétroviral chez les femmes enceintes VIH-1 positif, sur la transmission de l'infection de la mère à l'enfant: cas du Centre Médical Saint Camille de Ouagadougou, au Burkina Faso

**DOI:** 10.11604/pamj.2015.20.399.5627

**Published:** 2015-04-23

**Authors:** Serge Theophile Soubeiga, Rebecca Compaore, Florencia Djigma, Nicaise Zagre, Elsa Assengone, Lassina Traore, Birama Diarra, Cyrille Bisseye, Djeneba Ouermi, Tani Sagna, Simplice Karou, Virginio Pietra, Jacques Simpore

**Affiliations:** 1 Centre de Recherche Biomoléculaire Pietro Annigoni (CERBA), Laboratoire de Biologie Moléculaire et de Génétique (LABIOGENE), Université de Ouagadougou, Burkina Faso; 2 Département de Biologie, Faculté des Sciences Université des Sciences et Techniques de Masuku (USTM) BP 934, Franceville, Gabon; 3 Ecole Supérieure des Techniques Biologiques et Alimentaires (ESTBA-UL), Université de Lomé, Togo

**Keywords:** VIH-1, PTME, DBS, Burkina Faso, HIV-1, PMTCT, DBS, Burkina Faso

## Abstract

**Introduction:**

L'infection au VIH chez les nouveau-nés par leur mère peut être réduite grâce à des programmes de prévention de transmission mère-enfant du VIH (PTME). L'objectif dans cette étude était d’évaluer le traitement antirétroviral chez les femmes enceintes VIH-1 positif sur la transmission mère-enfant de l'infection au Centre Médical Saint Camille de Ouagadougou, Burkina Faso.

**Méthodes:**

Des échantillons de spot de sang total ont été collectés chez 160 enfants âgés de 6 semaines, nés de mères VIH-1 positif et chez 40 enfants âgés de 2 à 13 mois provenant d'orphelinats et dont les mères étaient inconnues. Ces échantillons ont été testés avec le kit Abbott Real Time HIV-1 Qualitative. Un questionnaire a permis de connaitre les âges et les fonctions des femmes enceintes.

**Résultats:**

Les femmes enceintes avaient un âge moyen global de 29,50±5,19 ans. Au total, 50,5% (101/200) ont été mises sous combinaison AZT/3TC/NVP et 29,5% (59/200) étaient sous prophylaxie (AZT/3TC). Le taux de transmission verticale du VIH-1 était de 0,0% (0/160) (p < 0,001) chez les enfants dont les mères étaient sous combinaison AZT/3TC/NVP ou sous prophylaxie AZT/3TC et de 15,0% (6/40) chez les enfants orphelins qui n’étaient pas inclus dans le protocole de la PTME.

**Conclusion:**

Selon les résultats, le protocole de la PTME est efficace et réduit très significativement le risque de transmission du VIH-1 de la mère à l'enfant. De plus, le dépistage par PCR, des enfants orphelins infectés verticalement par le VIH, permet leur prise en charge thérapeutique précoce.

## Introduction

Le nombre d'enfants infectés par an ne cessait d'augmenter jusqu'en 2002, année où il a atteint son niveau maximum, soit environ 590 000. En 2010, il était de 350 000 selon les estimations de l'OMS [1]. La Transmission mère-enfant du VIH-1 (TME/VIH-1) peut se faire in utero, pendant l'accouchement et après la naissance à travers l'allaitement maternel [2]. Le risque de transmission est de 5 à 10% pendant la grossesse, 10 à 20% pendant l'accouchement, de 5 à 10% durant les 6 premiers mois de l'allaitement et de 10 à 15% quand la période d'allaitement est étendue à 24 mois [3]. En absence de traitement, le risque combiné de TME/VIH in utero et intrapartum est de 15 à 30% et ce risque est augmenté chez l'enfant nourri au lait maternel de 20 à 45% [4]. Le risque de transmission du VIH à travers l'allaitement maternel est 3 à 10 fois plus élevé chez les femmes VIH-1 positif ayant un taux de lymphocytes CD4 inférieur à 200 cellules par millilitre de sang que chez celles qui ont un taux de CD4 supérieur à ce seuil [5]. La défaillance du système immunitaire constitue donc un facteur de risque de transmission du VIH de la mère à l'enfant.

Le traitement antirétroviral appliqué chez une femme VIH positif au début de sa grossesse, peut réduire de façon significative le risque de transmission de l'infection de la mère à l'enfant et peut cependant contribuer à la réduction du taux de transmission du VIH en Afrique subsaharienne. Le Conseil Dépistage (CD) est très nécessaire lors des consultations prénatales pour identifier les femmes enceintes infectées par le VIH et leur appliquer un protocole pour éviter une transmission probable du VIH à leur enfant pendant la grossesse, l'accouchement ou l'allaitement maternel. Aussi, le diagnostic de l'infection à VIH par PCR permet d'identifier les enfants infectés nés de mères VIH positif et de commencer un traitement précoce afin d’éviter le risque de mortalité. En effet, l'initiation précoce du Traitement Antirétroviral Hautement Actif (HAART) chez les enfants réduit de façon significative la morbidité et la mortalité associées au VIH [6, 7]. Au Burkina Faso, le protocole de Prévention de la Transmission Mère-Enfant (PTME) est en vigueur depuis 2002 au Centre Médical Saint Camille de Ouagadougou. Ce protocole vise à identifier les femmes enceintes et les mères infectées par le VIH et à réduire significativement le risque de transmission de l'infection de la mère à l'enfant. Il est appliqué chez les femmes qui ont donné leur consentement libre après counselling (Option In-Out). L'objectif de cette étude est d’évaluer le traitement antirétroviral chez les femmes enceintes infectées par le VIH-1 sur la transmission de l'infection de la mère à l'enfant au Centre Médical Saint Camille de Ouagadougou au Burkina Faso.

## Méthodes

**Type et cadre de l’étude:** Cette étude est de type transversal et s'est déroulée de 2012 à 2013 au Centre Médical Saint Camille de Ouagadougou, au Burkina Faso.

**Population d’étude:** Pour cette étude, 160 femmes enceintes dépistées VIH-1 positif au cours de leur consultation prénatale (CPN), ont été suivies depuis leur grossesse jusqu’à leur accouchement. Toutes ces femmes ont suivi le protocole de la PTME. Le diagnostic précoce de l'infection à VIH-1 par PCR en temps réel a été effectué chez les enfants, 6 semaines après leur naissance. Egalement, 40 autres enfants âgés de 2 à 13 mois provenant d'orphelinats, dont les mères décédées étaient VIH séropositives, ont été inclus dans cette recherche et dépistés par PCR en temps réel. Un questionnaire a permis de recueillir les âges et les fonctions de chaque femme ayant accepté librement de faire partie de cette étude.

**Traitement des femmes enceintes dépistées VIH-1 (+):** Au cours de leur consultation prénatale, les femmes enceintes ont subi un counselling et un dépistage. Les femmes enceintes VIH-1 positif ont été mises sous prophylaxie Zidovudine/Lamivudine (AZT/3TC) ou sous trithérapie Zidovudine/Lamivudine/Névirapine (AZT/3TC/NVP) sur la base de leur taux de CD4. Celles qui avaient un taux de CD4> 350 cellules/mm3 ont été placées sous Option A de l'OMS. Par contre, les femmes enceintes qui avaient un taux de CD4>350 cellules/mm3 ont été mises sous AZT/3TC/NVP. En cas de contre-indication pour la Névirapine (allergie, hépatopathies), elle est remplacée par l'Efavirenz (EFV) qui, est utilisé en dehors du premier trimestre de la grossesse.

**PCR qualitative en temps réel:** Des spots de sang total (DBS) ont été réalisés sur des papiers buvards Wattman à partir de prélèvements capillaires. Les échantillons obtenus (DBS) ont été conservés à température ambiante jusqu’à l'analyse par PCR en temps réel avec le kit Abbott Real Time HIV-1 Qualitative (promega, USA) dont la spécificité et la sensibilité sont respectivement de 100% et de 2500 copies/ml selon le fabricant.

**Comité d’éthique:** Le comité d’éthique institutionnel du Centre Médical Saint Camille/CERBA a donné son approbation pour la réalisation de l’étude et toutes les mères ont librement donné leur consentement sur la participation de leur enfant à l’étude.

**Analyses statistiques:** Les données ont été analysées sur Excel 2007 et ensuite sur les logiciels Statistical Package Social Sciences (SPSS) version17.0 et sur Epi Info version 6.0 pour le calcul du test de chi^2^. La valeur significative a été fixée à p < 0,001.

## Résultats

Ont été inclus, dans cette étude, 200 enfants dont 160 sont nés de mères VIH-1 positif qui ont suivi le protocole de la PTME et 40 enfants orphelins. La moyenne d’âge de ces 160 femmes enceintes était de 29,50 ± 5,19 ans. Et parmi elles, 75,00% (120/160) étaient des ménagères (Tableau 1). Dans cette étude, 50,50% (101/200) des mères étaient sous HAART, 29,50% (59/200) étaient sous traitement ARV de prophylaxie et 20,00% (40/200) n'avaient pas suivi le protocole de la PTME. Cette étude a montré que le taux de transmission verticale était de 0,0% (0/160) chez les enfants dont les mères étaient sous HAART (AZT/3TC/NVP) ou sous prophylaxie (AZT/3TC). Par contre chez les orphelins, la transmission était de 15,0% (6/40) (Tableau 2). Tous les 6 enfants détectés VIH positif ont été immédiatement pris en charge par le service de la pédiatrie pour un traitement antirétroviral spécialisé (AZT/3TC, NVP ou D4T/3TC).

**Tableau 1:** Ages et fonctions des femmes enceintes VIH-1 positif sous protocole de la PTME

N°	Fonction	Nombre	Pourcentage (%)	Age moyen (ans)
1	Ménagères	120	75,00	29,18 ± 5,17
2	Secteur informel	33	20,62	29,98 ± 5,22
3	Salariées	7	4,38	33,11 ± 4,62
Total		160	100	29,50 ± 5,19

1 → 2 : P< 0,001 1 → 3 : P< 0,001 2 → 3 : P= 0,427

**Tableau 2:** Traitement des mères VIH-1 positif et résultats des PCR des enfants

		Enfants		
Traitement (Mères)	Mères VIH-1 (+)	PCR Négative	PCR Positive	X2 test
HAART	101	101 (100%)	0 (0,00%)	
Prophylaxie	59	59 (100%)	0 (0,00%)	p < 0,001
Sans traitement	40	34 (85,00%)	6 (15,00%)	
Total	200	200 (7,00%)	6 (3,00%)	

## Discussion

Les femmes enceintes VIH-1 positif de cette étude était relativement jeune avec un âge moyen de 29,50 ± 5,19 et la majorité d'entre elles était des ménagères soit 75,00% (120/160). Les résultats de la transmission verticale trouvés dans cette étude chez les mères sous prophylaxie ou HAART et celles qui ne sont pas sous traitement témoignent l'efficacité de la Névirapine (NVP) et de la trithérapie dans la prévention de la transmission mère-enfant du VIH confirmée par certains auteurs tels Pignatteli et ses collaborateurs en 2006 [8] et ensuite par Simporé et ses collaborateurs en 2006 et en 2007 [9, 10]. Le taux de transmission de 0,0% trouvé chez les mères suivant le protocole de la PTME est très faible à celui trouvé par Linguissi et al. en 2012 [11] qui trouvaient un taux de transmission verticale globale de 4,8% par la technique de PCR en temps réel. Une étude menée par Kouanda et al. en 2010 [12] trouvait un taux de transmission verticale de 0,0% (0/195) et de 4,6% (12/259) respectivement chez les mères sous HAART et celles sous prophylaxie. La transmission de l'infection à VIH-1 chez les enfants (15,0%) dans cette étude, s'est produite probablement pendant la grossesse, l'accouchement ou l'allaitement maternel.

Un traitement (prophylaxie ou HAART) bien suivi par une femme enceinte ou bien une mère VIH positif réduit très significativement le risque de transmission du VIH à l'enfant contrairement à une femmes enceinte qui n'est pas sous protocole PTME. La prophylaxie avec un ou plusieurs ARV réduit le risque de transmission pendant l'accouchement [13] mais le risque de transmission postnatale reste élevé dans les milieux où l'allaitement maternel est pratiqué. Selon l'OMS, les femmes enceintes ayant un taux de CD4 < 350 cellules/mm^3^sont éligibles pour la trithérapie. Une fois initiée, le traitement antirétroviral hautement actif réduit rapidement et constamment la charge virale maternelle dans le plasma et dans le lait, réduisant probablement le risque de la transmission mère-enfant du VIH à travers l'allaitement maternel [14].

L'efficacité du protocole de la PTME réalisé dans cette étude vient démontrer l'avantage de débuter le traitement chez une femme VIH positif au début de sa grossesse. Mais l'inconvénient majeur est le développement d'une résistance acquise chez certaines mères et leurs enfants à la Névirapine après 6 mois de traitement, car la Névirapine induit des mutations sur le gène pol entraînant la résistance du VIH aux médicaments antirétroviraux [15, 16]. Plusieurs auteurs ont également confirmé que la Névirapine peut provoquer la substitution de la Valine en position 106 (V106I) sur la transcriptase inverse [17, 18]. 1/10^ème^ à 2/3 des femmes qui prennent une simple dose de NVP développeront une résistance virale aux inhibiteurs non-nucléosidiques de la transcriptase inverse (INNTIs) [19]. C'est pour donc éviter la résistance du VIH à la Névirapine, que l'OMS a préconise la combinaison AZT/3TC à la place de la NVP. Ce problème peut être considérablement réduit en combinant la NVP avec 3TC (± ZDV) pendant sept (7) jours après accouchement [20].

Une simple dose intrapartum de Ténofovir (TDF) et d'Emtricitabine (FTC) peut aussi réduire cette résistance du VIH aux INNTIs de moitié [21]. Selon les nouvelles recommandations de 2013, l'OMS préconise le traitement antirétroviral (TARV) à vie pour toute femme enceinte et allaitante infectée par le VIH quel qu'en soit le stade clinique ou le nombre de CD4. Le traitement doit être initié et maintenu après accouchement et cessation de l'allaitement (Option B+). Pour les femmes enceintes et allaitantes qui ne sont pas éligibles au traitement (CD4>500 cellules/mm^3^), le traitement doit être initié et arrêté après accouchement et cessation de l'allaitement. Pour les femmes développant un échec thérapeutique pendant leur grossesse ou pendant la période d'allaitement, le traitement de seconde ligne doit être imposé. Dans le cas des femmes allaitantes, le TARV doit être arrêté une semaine après la cessation de l'allaitement; et en cas d'allaitement artificiel, le TARV doit être arrêté après l'accouchement (Option B) [22]. Dans le but de réduire la transmission mère-enfant du VIH en Afrique, il est d'une grande importance que des efforts soient faits pour que toute femme enceinte dépistée VIH positif suive le protocole de la PTME.

## Conclusion

La transmission verticale nulle trouvée chez les enfants dont les mères étaient sous traitement (prophylaxie ou HAART), montre que le protocole de Prévention de la transmission mère-enfant réduit très significativement (p< 0,001) le risque de transmission du VIH d'une mère VIH positif à son enfant. Toutefois, il est important de rester vigilant car une inattention pourrait augmenter ce taux. Malgré ces résultats, beaucoup d'efforts restent à faire pour augmenter la couverture du protocole de la PTME en Afrique et plus particulièrement au Burkina Faso.
